# Co-Crystal Screening of Diclofenac

**DOI:** 10.3390/pharmaceutics3030601

**Published:** 2011-08-31

**Authors:** Christer B. Aakeröy, Angela B. Grommet, John Desper

**Affiliations:** Department of Chemistry, Kansas State University, Manhattan, KS 66506, USA

**Keywords:** diclofenac, co-crystals, hydrogen bonding, crystallography, IR spectroscopy

## Abstract

In the pharmaceutical industry, co-crystals are becoming increasingly valuable as crystalline solids that can offer altered/improved physical properties of an active pharmaceutical ingredient (API) without changing its chemical identity or biological activity. In order to identify new solid forms of diclofenac—an analgesic with extremely poor aqueous solubility for which few co-crystal structures have been determined—a range of pyrazoles, pyridines, and pyrimidines were screened for co-crystal formation using solvent assisted grinding and infrared spectroscopy with an overall success rate of 50%. The crystal structures of three new diclofenac co-crystals are reported herein: (diclofenac)·(2-aminopyrimidine), (diclofenac)·(2-amino-4,6-dimethylpyrimidine), and (diclofenac)·(2-amino-4-chloro-6-methylpyrimidine).

## 1. Introduction

Co-crystallization offers the supramolecular chemist a unique opportunity—it provides the ability to tune a compound's physical properties without altering its chemical identity. While this technique is gaining momentum in applications ranging from materials science to agro-chemistry, the field in which co-crystallization has progressed farthest is arguably the pharmaceutical industry. In recent years [1], pharmaceutical co-crystals have been developed such that several physical properties of the co-crystal—solubility [2], bioavailability, thermal stability, *etc.*—are improved compared to those of the pure drug [3,4]. In pharmaceutical co-crystals, hydrogen bonding between carboxylic acids and aromatic nitrogen atoms has proven particularly reliable [5]. For example, Lemmerer *et al.* recently reported a (terephthalic acid)·(isonicotinic acid hydrazide) pharmaceutical co-crystal [5] using this particular supramolecular synthon [6].

**Scheme 1.**
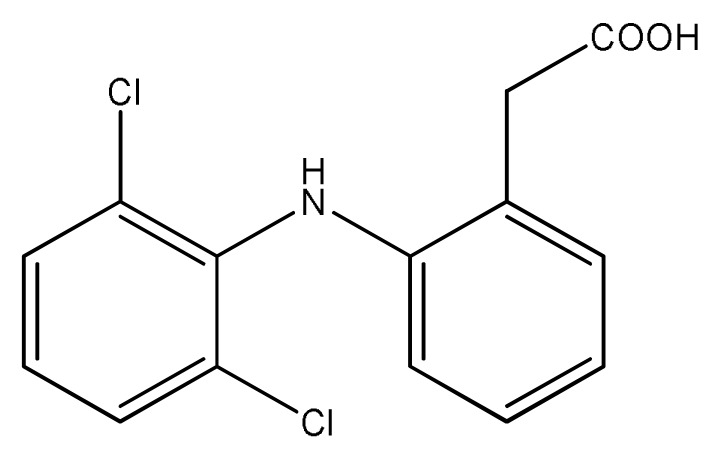
Diclofenac.

Diclofenac—a potent, non-steroidal, anti-inflammatory analgesic with extremely poor aqueous solubility (0.9 ± 0.1 μg mL^−1^) [7]—also possesses a carboxylic acid, suggesting that this API might be favorable to similar co-crystallizations. Indeed, Báthori *et al.* did show that the carboxylic acid in diclofenac interacts favorably with the aromatic nitrogen atom in isonicotinamide [8]. However, there have been no reported studies that have systematically explored (a) the ability of diclofenac to form co-crystals and (b) which structural motifs diclofenac is most likely to generate. As diclofenac itself has two hydrogen bond donors, three hydrogen bond acceptors, and significant conformational flexibility, Scheme 1, it is important to map out the intermolecular landscape that this molecule inhabits. In this study, we establish the propensity of diclofenac to co-crystallize with compounds containing aromatic nitrogen atoms. Initially, infrared spectroscopy was used to probe the reaction of diclofenac with compounds from an extensive list of pyrazoles, pyridines, and pyrimidines spanning a wide range of charges on the aromatic nitrogen. The results from this screen as well as the crystal structures of three new co-crystals of diclofenac are presented in this study.

## 2. Experimental Section

### 2.1. Materials

2-Aminopyrimidine and 2-amino-4,6-dimethylpyrimidine were purchased from Acros Organics. 2-Amino-4-chloro-6-methylpyrimidine was purchased from Aldrich. Sodium diclofenac was purchased from TCI America.

### 2.2. Preparation of Diclofenac

3.00 g (9.43 mmol) of sodium diclofenac was dissolved in a minimum amount of water. 1 M HCl was added dropwise to the solution until the reaction was complete or until no more diclofenac was precipitating. The product was filtered, dried, and dissolved in a minimum amount of ethanol. The ethanol solution was covered with parafilm and placed in the freezer (0 °C) to encourage the re-crystallization of the product (90% yield).

### 2.3. Co-Crystal Screening

Diclofenac and its prospective co-crystallizing agents were combined *via* solvent assisted grinding (SAG). 0.010 g (0.034 mmol) of diclofenac and an equimolar amount of the co-crystallizing agent were combined in a mortar. After the addition of 1 drop of methanol, the mixture was ground with a pestle until dry. This process was repeated twice. The samples produced from SAG were analyzed using infrared spectroscopy. The IR used was a Thermo Scientific Nicolet 380 FT-IR Spectrometer with a zinc selenide ATR crystal. On occasion, melt experiments were used to verify results from SAG. 0.010 g (0.034 mmol) of diclofenac and an equimolar amount of the co-crystallizing agent were combined in a test tube. The test tube was then heated with a heat gun until both components were melted. Samples were cooled and analyzed using infrared spectroscopy.

### 2.4. Co-Crystal Synthesis

Single crystals of the diclofenac co-crystals were grown using vapor diffusion (VD). In VD, equimolar amounts of each component are combined in a vial and dissolved in an appropriate solvent. The open vial is placed upright in a sample jar filled 2 cm deep with a second solvent—both components should be poorly soluble in this second solvent, but the two solvents must be miscible with each other. The lid of the sample jar is then fastened securely and sealed with parafilm.

#### 2.4.1. Synthesis of (diclofenac)·(2-aminopyrimidine), (DC)·(AP)

0.010 g (0.034 mmol) of diclofenac and 0.0032 g (0.034 mmol) of 2-aminopyrimidine were combined in a vial and dissolved in a minimum amount of chloroform, with hexanes filling the bottom of the sample jar. After three weeks, co-crystals formed as colorless, transparent blocks approximately 0.3 × 0.2 × 0.1 cm, m.p. 168–172 °C.

#### 2.4.2. Synthesis of (diclofenac)·(2-amino-4,6-dimethylpyrimidine), (DC)·(AMP)

0.010 g (0.034 mmol) of diclofenac and 0.0049 g (0.034 mmol) of 2-amino-4,6-dimethylpyrimidine were combined in a vial and dissolved in a minimum amount of ethyl acetate, with hexanes filling the bottom of the sample jar. After three weeks, co-crystals formed as colorless, transparent, cubic crystals with edges approximately 0.1 cm long, m.p. 173–174 °C.

#### 2.4.3. Synthesis of (diclofenac)·(2-amino-4-chloro-6-methylpyrimidine), (DC)·(ACMP)

0.010 g (0.034 mmol) of diclofenac and 0.0049 g (0.034 mmol) of 2-amino-4-chloro-6-methylpyrimidine were combined in a vial and dissolved in a minimum amount of ethyl acetate, with hexanes filling the bottom of the sample jar. After three weeks, co-crystals formed as colorless, transparent rods approximately 0.75 cm long, m.p. 169–172 °C.

### 2.5. Single Crystal X-Ray Crystallography

X-ray data were collected on a Bruker Kappa APEX II CCD diffractometer at 120 K using a fine-focus molybdenum Kα tube. Data were collected using APEX [9] software. Initial cell constants were found by small widely separated “matrix” runs. Scan speed and scan width were chosen based on scattering power and peak rocking curves.

Unit cell constants and orientation matrix were improved by least-squares refinement of reflections thresholded from the entire dataset. Integration was performed with SAINT [10] using this improved unit cell as a starting point. Precise unit cell constants were calculated in SAINT from the final merged dataset. Lorenz and polarization corrections were applied. Laué symmetry, space group, and unit cell contents were found with XPREP [11].

Data were reduced with SHELXTL [12]. The structures were solved in all cases by direct methods without incident. Except as noted below, hydrogen atoms were assigned to idealized positions and were allowed to ride. Heavy atoms were refined with anisotropic thermal parameters. Multi-scan absorption correction was carried out on all datasets.

#### (DC)·(AP)

Coordinates of the carboxylic acid proton H(11), the amine proton H(12), and amine protons H(32A) and H(32B) were allowed to refine.

#### (DC)·(AMP)

Coordinates of the carboxylic acid proton H(11), the amine proton H(12), and amine protons H(32A) and H(32B) were allowed to refine. An extinction correction was applied to the dataset. The R_1_ and *w*R_2_ values for this compound are high because the crystal was a poor scatterer.

#### (DC)·(ACMP)

An alternate position for the halopyrimidine, with the chlorine and methyl groups changing places, was found in the difference electron density map. Population of this species refined to about 20% of total. Its geometry was restrained to that of the major species by means of the SHELX “SAME” command. Anisotropic thermal parameters for closely located atoms on the two species were pairwise constrained using the SHELX “EADP” command. Coordinates of the carboxylic acid proton H(11), the amine proton H(12), and amine protons H(32A) and H(32B) on the major species were allowed to refine; all other hydrogen atoms were given idealized coordinates.

### 2.6. Melting Point Determination

Melting points of solved co-crystal samples were determined using a Fisher-Johns Melting Point Apparatus and are uncorrected.

### 2.7. Cambridge Structural Database Search

The Cambridge Structural Database—Version 5.32 [13] with the November 2010 update—was used to search for existing diclofenac co-crystals. Neutral diclofenac was used as the search fragment, with the search parameters being 3D coordinates determined, *R* factor ≤0.075, not disordered, no errors, not polymeric, and only organics. 11 hits were obtained—elimination of eight crystal structures in which diclofenac was the only component yielded three remaining structures of diclofenac co-crystals.

### 2.8. Charge Calculation

The Spartan 2006 program was used to calculate the charges on the aromatic nitrogens of the co-crystallizing agents used in the co-crystal screen. The program was set to calculate Equilibrium Geometry at Ground state with Semi-Empirical AM1, Starting from Initial geometry, Subject to Symmetry, Total Charge: Neutral, and Multiplicity: Singlet. The Global Calculations box was checked. Under Surfaces, surface: density with property: potential was added.

## 3. Results and Discussion

### 3.1. Results of Co-Crystal Screening

Using SAG and IR, eighteen compounds from three different families—pyrazoles, pyridines, and pyrimidines—were screened for their ability to co-crystallize with diclofenac. The results of this screen are shown in Table 1, in which the target ligands are arranged in order of decreasing charge magnitude.

Three different types of interactions were observed. In salt formation (salt), the carbonyl peak in the original neutral diclofenac has been converted into a carboxylate moiety, COO^−^, which appears in the IR spectrum below 1675 cm^−1^. The primary hydrogen bond between the two components, O⋯H– N(heterocycle), generates two broad stretches at approximately 1900 cm^−1^ and 2500 cm^−1^. Co-crystallization (+) also yields two broad stretches at approximately 1900 cm^−1^ and 2500 cm^−1^, indicative of O–H⋯N(heterocycle) hydrogen bonds, accompanied by a smaller shift of the carbonyl peak on the IR spectra. When the two components do not react (−), a composite of their individual IR spectra is produced.

Of the eighteen potential co-crystallizing agents listed in Table 1, two compounds formed salts, nine compounds formed co-crystals, and the remaining seven compounds did not react with diclofenac. None of the pyrazoles reacted in any way; whereas 83% of the pyrimidines produced co-crystals with diclofenac. Finally, all pyridines reacted with diclofenac—33% formed salts and 66% formed neutral co-crystals.

### 3.2. Results of Single-Crystal X-Ray Diffraction Studies

From the list of co-crystallizing agents presented in Table 1, three structures of diclofenac and 2-aminopyrimidines were determined using single crystal X-ray diffraction, Table 2. All three structures—(DC)·(AP), (DC)·(AMP), and (DC)·(ACMP)—are remarkably similar.

#### 3.2.1. Structure of (diclofenac)·(2-aminopyrimidine), (DC)·(AP)

**Figure 1.**
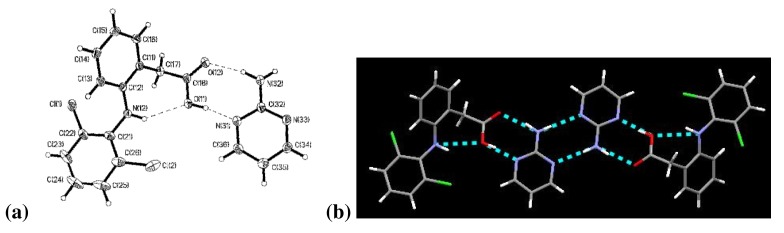
**(a)** (DC)·(AP); **(b)** Tetramer of (DC)·(AP).

The two aromatic rings of diclofenac are bent out of plane, with a torsion angle of 57.7°. Connecting these aromatic rings is an –NH moiety in which the nitrogen atom, N(12), exhibits trigonal pyramidal molecular geometry. The carboxylic acid proton, H(11), is covalently bonded to diclofenac, which establishes the structure as a co-crystal instead of a salt, Figure 1a. Indeed, the bond lengths between C(18)–O(11) and C(18)–O(12) are 1.3168(15) Å and 1.2187(14) Å, respectively, indicative of neutral C–O and C=O bonds. This result is consistent with the assignment from the IR work and underscores that the acidic proton has not been transferred to 2-aminopyrimidine.

Adjacent molecules of diclofenac and 2-aminopyrimidine are assembled into tetrameric supermolecules in a 1:1 stoichiometry connected *via* four hydrogen bonds, Figure 1b. There are three unique intermolecular hydrogen bonds: between O(12)⋯N(32), between O(11)⋯N(31), and between amine and aromatic nitrogen in the 2-point interaction of the 2-aminopyrimidines, Table 3. There is also an intramolecular hydrogen bond N(12)⋯O(11).

#### 3.2.2. Structure of (diclofenac)·(2-amino-4,6-dimethylpyrimidine), (DC)·(AMP)

**Figure 2.**
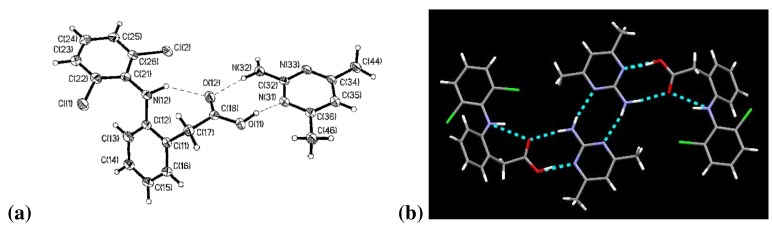
**(a)** (DC)·(AMP); **(b)** Tetramer of (DC)·(AMP).

The two aromatic rings of diclofenac are twisted by 59.2° with respect to each other. Connecting these aromatic rings is the –NH group in which the nitrogen atom, N(12), exhibits trigonal pyramidal molecular geometry. The carboxylic acid proton, H(11), is again covalently bonded to the carboxylic acid group on diclofenac, Figure 2a. The bond lengths C(18)–O(11) and C(18)–O(12) are 1.302(4) Å and 1.223(4) Å, respectively.

Diclofenac and 2-amino-4,6-dimethylpyrimidine are present in a 1:1 stoichiometry and are arranged in a discrete tetrameric motif connected *via* four hydrogen bonds. There are three unique intermolecular hydrogen bonds: between O(12)⋯N(32), between O(11)⋯N(31), and between amine and aromatic nitrogen atom in the 2-point interaction of the 2-amino-4,6-dimethylpyrimidines, Figure 2b. There is also an intramolecular interaction between N(12)⋯O(12).

#### 3.2.3. Structure of (diclofenac)·(2-amino-4-chloro-6-methylpyrimidine), (DC)·(ACMP)

**Figure 3.**
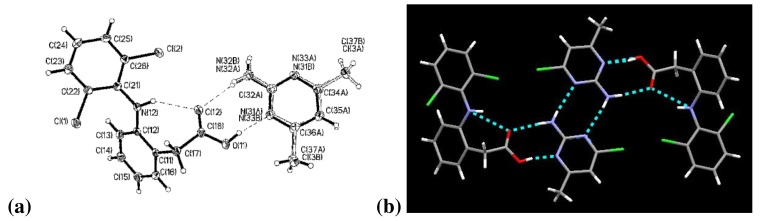
**(a)** (DC)·(ACMP); **(b)** Tetramer of (DC)·(ACMP).

The two aromatic rings of diclofenac are bent out of plane, with a torsion angle of 59.2°. Connecting these aromatic rings is an –NH in which the nitrogen atom, N(12), exhibits trigonal pyramidal molecular geometry, Figure 3a. The carboxylic acid proton, H(11), remains covalently bonded to diclofenac and the C(18)–O(11) and C(18)–O(12) bond lengths are 1.3164(17) Å and 1.2204(16) Å, respectively.

Diclofenac and 2-amino-4-chloro-6-methylpyrimidine are present in a 1:1 stoichiometry and are assembled *via* four hydrogen bonds into a tetrameric supermolecule. Three are intermolecular hydrogen bonds: between O(12)⋯N(32A) or O(12)⋯N(32B), between O(11)⋯N(31A), and a 2-point interaction between amine and aromatic nitrogen in the 2-point interaction of the 2-amino-4-chloro-6-methylpyrimidines, Figure 3b. The fourth hydrogen bond is an intramolecular bond between N(12)⋯O(12). The alternate position for the halopyrimidine, with the chlorine and methyl groups changing places, leads to the additional hydrogen bonds shown for (DC)·(ACMP) in Table 3.

### 3.3. Discussion

#### 3.3.1. Discussion of Co-Crystal Screening

A broad range of pyrazoles, pyridines, and pyrimidines have been screened for co-crystal formation with an overall success rate of 50% despite the fact that re-crystallization (resulting in homomeric solids) is usually much more favorable than co-crystallization [14]. As seen in Table 1, two of the compounds with the most negatively charged nitrogens produced salts with diclofenac. In both cases, the charge on these nitrogens was great enough to cause the acidic proton to leave diclofenac and bond covalently with pyridine, initiating the bonding between the anionic diclofenac and the pyridinium ions.

Unfortunately, the charge boundary differentiating salt from co-crystal is not well defined. While the aromatic nitrogen atoms on 2-aminopyridine and 2-amino-4,6-dimethylpyrimidine both display a charge of −287 kJ/mol, 2-aminopyridine is thought to be a salt, and 2-amino-4,6-dimethylpyrimidine has been determined to be a co-crystal. Initially, both compounds were thought to form salts with diclofenac—2-aminopyridine and 2-amino-4,6-dimethylpyrimidine were both screened using SAG with methanol (a polar protic solvent) with carboxylate peaks appearing on the IR spectra at 1670 cm^−1^ and 1664 cm^−1^, respectively. When single crystals of (diclofenac)·(2-amino-4,6-dimethylpyrimidine) were grown from ethyl acetate (a polar aprotic solvent), the IR yielded a similar value: 1695 cm^−1^. The structure of (DC)·(AMP), however, showed it to be a co-crystal. Melt experiments were then performed to investigate the interactions of 2-aminopyridine and 2-amino-4,6-dimethylpyrimidine with diclofenac in the absence of solvent affects. The carboxylate peak for the 2-aminopyridine and diclofenac sample occurred well below the 1675 cm^−1^ salt/co-crystal barrier, consistent with the results from SAG. In the sample containing 2-amino-4,6-dimethylpyrimidine and diclofenac, however, the carbonyl peak occurred at 1682 cm^−1^, above the salt/co-crystal boundary and in agreement with the structural evidence for co-crystal formation.

The next seven compounds down the list of charge also reacted favorably with diclofenac, forming co-crystals. In these cases, the aromatic nitrogen atoms were characterized by intermediate charge magnitude—too weak to break the covalent bond of diclofenac's acid proton, they were nevertheless powerful enough to form stable hydrogen bonds with carboxylic acid and to break the acid⋯acid dimer in neutral diclofenac. Of the remaining compounds, most did not react with diclofenac—they were too electrostatically unattractive to form any type of bond with diclofenac.

There was, however, one exception to the trend exhibited in Table 1. 2-Amino-3,5-dibromopyridine has one of the most weakly charged aromatic nitrogen atoms on the list. Nevertheless, the IR indicated that it formed a co-crystal with diclofenac. This seeming anomaly can be justified by examining the structure of 2-amino-3,5-dibromopyridine. While the charge on the aromatic nitrogen may place this compound firmly among poor hydrogen bond acceptors, the amine is a hydrogen bond donor fully capable of forming stable hydrogen bonds with the carboxylic acid on diclofenac. The tendency of amines to interact with a carboxylic acid is well demonstrated in Table 1—all compounds containing this functional group react successfully with diclofenac.

#### 3.3.2. Discussion of X-Ray Diffraction results

**Scheme 2.**
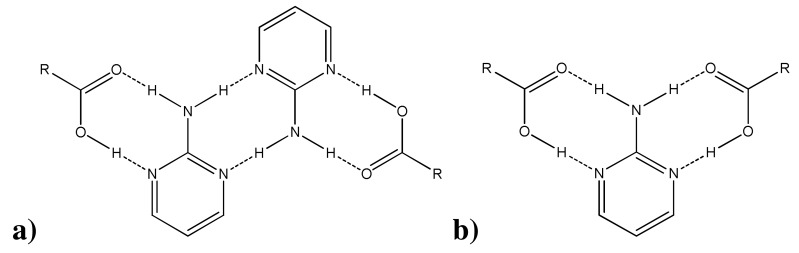
**a)** homomeric interaction **b)** heteromeric interaction.

Based on existing literature reports, [15,16], (carboxylic acid)·(2-aminopyrimidine) co-crystals have been observed to organize themselves according to two general patterns—homomerically or heteromerically [17]—as seen in Scheme 2. CSD version 5.32 with the November 2010 update was used to probe the frequency of these interactions. 2-Aminopyrimidine and carboxylic acid was used as the search fragment, and the search parameters were 3D coordinates determined, *R* factor ≤0.1, not disordered, no errors, not polymeric, no ions, no powder structures, and only organics. Out of 53 co-crystal hits, 39 were arranged homomerically or heteromerically—the remaining 14 co-crystals were organized neither according to these general formations nor in other easily recognized patterns. Of the 39 homomeric/heteromeric hits, homomeric interactions were slightly more prevalent, forming at a rate of 56%. In all three co-crystal structures reported here, a 1:1 stoichiometry was observed. Given our previous knowledge of the binding patterns of (carboxylic acid)·(2-aminopyrimidine) co-crystals, these structures would be expected to further exhibit one of the binding patterns in Scheme 2—as indeed they do. All three (diclofenac)·(2-aminopyrimidine) co-crystals are organized homomerically.

In addition to bonding homomerically, two of the co-crystal structures—(D)·(AMP) and (D)·(ACMP)—exhibit borderline Type 1 halogen bonding [18]. For (D)·(AMP), *θ*_1_ = *θ*_2_ = 155.87°. For (D)·(ACMP), *θ*_1_ = *θ*_2_ = 156.45°. While *θ*_1_ and *θ*_2_ should be close to 120° by definition, the halogen bonding angles for both structures are greater than this optimal angle by more than 30°. Nevertheless, this contact organizes both structures into infinite, 1D chains—a continuous sequence of homomerically bonded units.

The opportunity to tune an API's physical properties without altering its chemical identity is a significant incentive for growing pharmaceutical co-crystals. While physical properties such as solubility, bioavailability, and thermal stability command interest from the pharmaceutical industry, they are time consuming to quantitatively establish. The melting point of a co-crystal, however, is a physical property which is relatively easy to determine, and comparing the melting points of co-crystallizing agent, co-crystal, and API often reveals if a particular co-crystal has the potential to affect physical properties of greater pharmaceutical relevance [19]. As seen in Table 4, the melting points of the salts and co-crystals discovered in this screen indicate that these new compounds do indeed have the capacity to alter the physical properties of diclofenac.

**Figure 4.**
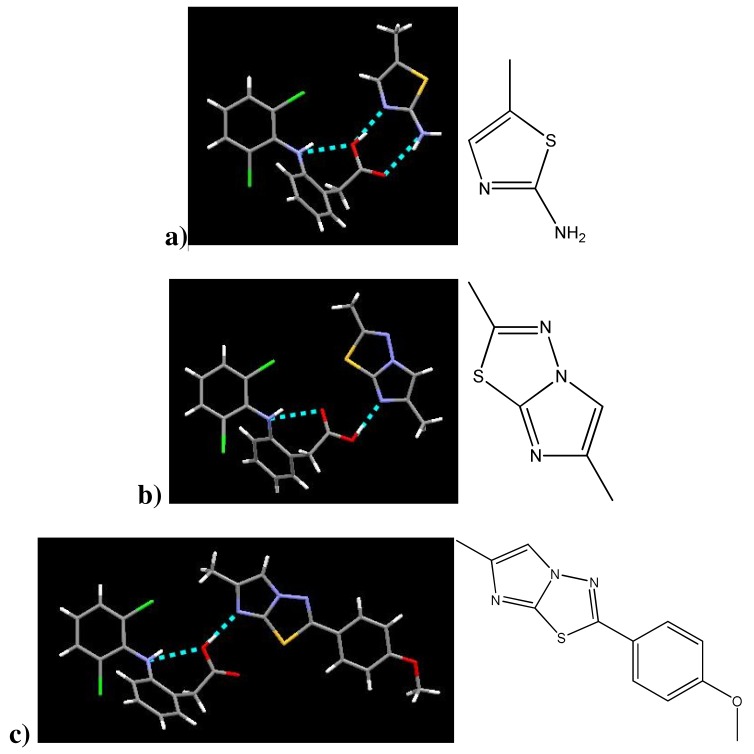

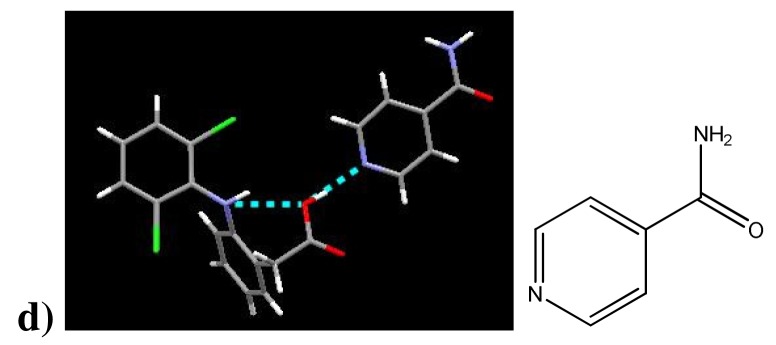
(**a**) co-crystal of diclofenac and 2-amino-5-methyl-1,3-thiazolium (DC)·(1); (**b**)co-crystal of diclofenac and 2,6-dimethylimidazo[2,1-b][1,3,4]thiadiazole (DC)·(2); (**c**)co-crystal of diclofenac and 2-(4-methoxyphenyl)-6-methylimidazo[2,1-b][1,3,4] thiadiazole (DC)·(3); (**d**) co-crystal of diclofenac and isonicotinamide (DC)·(4).

Three structures of existing diclofenac co-crystals are available on the CSD [20]. One other diclofenac co-crystal structure not yet available on the CSD—(diclofenac)·(isonicotinamide)—has been published [8]. The co-crystallizing agents for these co-crystals are shown in Figure 4. All four of these co-crystals provide good evidence for the affinity between carboxylic acids and aromatic nitrogen atoms.

Diclofenac interacts with (1) *via* a 2-point hydrogen bond with the aromatic nitrogen and the amine. In b), diclofenac forms a hydrogen bond with the aromatic nitrogen in the ring lacking the sulfur. The same interaction takes place in the co-crystal (DC)·(3). Diclofenac interacts with (4) both at the aromatic nitrogen and the amine. In all these structures the two aromatic rings of diclofenac are bent out of plane, the nitrogen atom of the –NH connecting these rings exhibits trigonal pyramidal molecular geometry, and the carboxylic acid proton remains covalently bonded to diclofenac— observations which agree with those made of the (diclofenac)·(2-aminopyrimidine) co-crystal structures, Table 5. Also, the diclofenac –NH participates in intramolecular hydrogen bonding with the carboxylic acid as in our structures. Lacking the symmetry of the 2-aminopyrimidines and therefore unable to bind homomerically, co-crystallizing agents (1)-(3) nevertheless form 1:1 co-crystals. Diclofenac and isonicotinamide co-crystallize in a 2:1 ratio. There is no suggestion of intermolecular halogen bonding in any of these co-crystal structures.

## 4. Conclusions

Eighteen compounds—pyrazoles, pyridines, and pyrimidines—with a wide range of charge on their aromatic nitrogen atoms were examined for co-crystal formation with diclofenac. These compounds successfully co-crystallized with diclofenac at a rate of 50%. The two compounds containing aromatic nitrogen atoms with the greatest charge magnitude formed salts with diclofenac; eight compounds with intermediate charge magnitude on their aromatic nitrogen atoms formed co-crystals with diclofenac; and seven of the compounds with aromatic nitrogen atoms of lowest charge magnitudes did not react with diclofenac. From the results of this screen, VD experiments were set up, and three structures of diclofenac co-crystals were determined: (DC)·(AP), (DC)·(AMP), and (DC)·(ACMP). These co-crystals were shown to alter the melting point of diclofenac, suggesting that physical properties of greater pharmaceutical relevance—solubility, bioavailability, thermal stability, *etc*.—have been altered. In this series of compounds, however, no direct correlation between melting points of either individual component and that of the co-crystal could be found. This is in contrast to previous studies [18] where the melting point of the co-crystallizing agents (a series of dicarboxylic acids) could be used to accurately predict the melting point of the corresponding co-crystals of bipyridine-based compounds. In those structures, the assemblies were typically arranged into infinite 2-D layers, with very limited conformational flexibility and variability. In the co-crystals presented herein, however, the primary tetrameric supermolecules are not linked into larger structural motifs and they have therefore much more freedom to pack in less consistent ways. This would suggest that in order to obtain molecular solids with tunable physical properties, it is important to limit the structural freedom of individual molecules and supermolecules and instead employ a design that leads to 2-D or 3-D motifs. We are currently exploring this hypothesis by co-crystallizing diclofenac with compounds that have the capability of creating infinite assemblies instead of discrete multimeric building blocks. We are also examining the influence that different solvents have on the outcome of the IR results and the structural pattern of co-crystals from this screen.

## Figures and Tables

**Table 1.** Co-Crystal Screening Results.

Compound	Charge (kJ/mol)	COO^−^ (cm^−1^)	C=O (cm^−1^)	O–H⋯N (cm^−1^)	Outcome
				*O⋯H–N (cm^−1^)	
3-aminopyridine*	−289	1654	n/a	2615, 2092	salt
2-aminopyridine*	−287	1670	n/a	2524, 1969	salt
2-amino-4,6-dimethylpyrimidine	−287	n/a	1665	2496, 1907	+
2-amino-4-chloro-6-methylpyrimidine	−278	n/a	1686	2529, 1904	+
3-hydroxypyridine	−276	n/a	1693	2533, 1904	+
2-amino-4-hydroxy-6-methylpyrimidine	−273	n/a	1697	2590, 1899	+
2-aminopyrimidine	−272	n/a	1697	2374, 1887	+
4,4′-bipyridine	−268	n/a	1704	2398, 1916	+
2-amino-5-chloropyridine	−267	n/a	1693	2566, 1985	+
4-chloro-2,6-diaminopyrimidine	−263	n/a	1683	2361, 1916	+
3,5-dimethylpyrazole	−256	n/a	1690	n/a	−
2-chloropyrimidine	−256	n/a	1691	n/a	−
Pyrazole	−254	n/a	1691	n/a	−
3,5-dimethyl-4-chloropyrazole	−246	n/a	1691	n/a	−
4-iodo-3,5-dimethylpyrazole	−240	n/a	1691	n/a	−
2-amino-3,5-dibromopyridine	−238	n/a	1696	2378, 1875	+
4-bromopyrazole	−228	n/a	1691	n/a	−
4-iodopyrazole	−227	n/a	1691	n/a	−
					

**Table 2.** Crystallographic Information.

	**(DC)**·**(AP)**	**(DC)**·**(AMP)**	**(DC)**·**(ACMP)**
Formula moiety	(C_14_H_11_NO_2_Cl_2_), (C_4_H_5_N_3_)	(C_14_H_11_NO_2_Cl_2_), (C_6_H_9_N_3_)	(C_14_H_11_NO_2_Cl_2_), (C_5_H_6_ClN_3_)
Empirical formula	C_18_H_16_C_l2_N_4_O_2_	C_20_H_20_Cl_2_N_4_O_2_	C_19_H_17_Cl_3_N_4_O_2_
Molecular weight	391.25	419.30	439.72
Color, Habit	colourless prism	colourless cube	colourless rod
Crystal system	triclinic	monoclinic	Monoclinic
Space group	P-1	P2(1)/c	P2(1)/c
Z	2	4	4
*a*, Å	7.4961(7)	22.270(2)	22.3435(14)
*b*, Å	8.7028(8)	4.6992(5)	4.7003(3)
*c*, Å	13.8697(12)	20.494(2)	20.4210(14)
*α*, °	76.747(2)		
*β*,°	87.392(3)	114.562(7)	115.092(3)
*γ*,°	86.408(2)		
Volume, Å^3^	878.54(14)	1950.6(4)	1942.2(2)
Crystal dimensions	0.28 × 0.22 × 0.14 mm	0.28 × 0.08 × 0.04 mm	0.22 × 0.12 × 0.08 mm
X-ray wavelength	0.71073	0.71073	0.71073
*μ*, mm^−1^	0.391	0.357	0.496
Absorption corr	multi-scan	multi-scan	multi-scan
R_int_	0.0361	0.0952	0.0571
Trans min/max	0.8985 / 0.9473	0.9066 / 0.9859	0.8988 / 0.9614
Reflections			
collected	27648	19020	35367
independent	6204	5670	7404
observed	4910	3166	5098
Threshold expression	>2σ(I)	>2σ(I)	>2σ(I)
R_1_ (observed)	0.0406	0.0787	0.0427
*w*R_2_ (all)	0.1072	0.2152	0.1059
Goodness of fit	1.088	1.114	1.097

**Table 3.** Hydrogen Bonds in Diclofenac Co-Crystals.

	**Bond length, D-H (Å)**	**Bond length, H⋯A (Å)**	**Bond length, D⋯A (Å)**	**Bond angle, D-H⋯A (°)**
**(DC)·(AP)**				
O(11) H(11) N(31)	0.940(18)	1.682(18)	2.6130(14)	170.2(17)
N(12) H(12) O(11)	0.873(17)	2.305(17)	3.0510(13)	143.5(14)
N(32) H(32A) O(12)	0.931(18)	2.000(19)	2.9191(15)	168.7(16)
N(32) H(32B) N(33)	0.876(18)	2.153(19)	3.0286(15)	176.9(17)
**(DC)·(AMP)**				
O(11) H(11) N(31)	0.98(4)	1.68(4)	2.642(3)	169(4)
N(12) H(12) O(12)	1.00(4)	2.11(4)	2.957(3)	142(3)
N(32) H(32A) O(12)	0.94(4)	2.13(4)	3.071(4)	172(3)
N(32) H(32B) N(33)	0.86(4)	2.27(4)	3.111(4)	166(3)
**(DC)·(ACMP)**				
O(11) H(11) N(31A)	0.866(17)	1.830(18)	2.688(5)	170.7(17)
N(12) H(12) O(12)	0.812(16)	2.275(17)	2.9793(16)	145.4(16)
N(32A) H(32A) O(12)	0.85(2)	2.22(2)	3.045(7)	163.4(18)
N(32A) H(32B) N(33A)	0.76(2)	2.44(2)	3.193(8)	172(2)
N(32B) H(32C) O(12)	0.88	2.29	3.15(3)	167.0
N(32B) H(32D) N(33A)	0.88	2.26	3.13(2)	170.8

**Table 4.** Melting Point Comparison.

Salt (*) or Co-crystal	M.P.of Co-crystallizing Agent	M.P.of Salt(*)or Co-crystal	M.P.of Diclofenac
(DC)·(3-aminopyridine)*	55–58 °C	108–112°C*	171–173 °C
(DC)·(2-aminopyridine)*	47–50 °C	73–74 °C*	171–173 °C
(DC)·(AMP)	151–153 °C	173–174 °C	171–173 °C
(DC)·(ACMP)	182–183 °C	169–172 °C	171–173 °C
(DC)·(3-hydroxypyridine)	126–129 °C	153–156 °C	171–173 °C
(DC)·(2-amino-4-hydroxy-6-methylpyrimidine)	>300 °C	>160 °C [sublimes]	171–173 °C
(DC)·(AP)	125–127 °C	168–172 °C	171–173 °C
(DC)·(4,4′-bipyridyl)	108–111 °C	155–157 °C	171–173 °C
(DC)·(2-amino-5-chloropyridine)	132–135 °C	150–154 °C	171–173 °C
(DC)·(4-chloro-2,6-diaminopyrimidine)	196–199 °C	155–160 °C	171–173 °C
(DC)·(2-amino-3,5-dibromopyridine)	98–100 °C	145–150 °C	171–173 °C

**Table 5.** Torsion Angles for Diclofenac Co-crystals.

**Co-crystal**	**Atoms selected**	**Torsion angle (°)**
(DC)·(AP)	C(22) C(21) C(12) C(13)	57.7
(DC)·(AMP)	C(22) C(21) C(12) C(13)	59.2
(DC)·(ACMP)	C(22) C(21) C(12) C(13)	59.2
(DC)·(1)	C(10) C(5) C(11) C(16)	−69.3
(DC)·(2)	C(12) C(7) C(13) C(18)	62.6
(DC)·(3)	C(18) C(13) C(19) C(24)	−61.7
(DC)·(4)	C(20) C(15) C(8) C(9)	73.5

## Supplementary Files

Supplementary File 1 CIF-Document (CIF, 53 KB)

## References

[1] Childs S., Zaworotko M. (2009). The Reemergence of Cocrystals: The Crystal Clear Writing Is on the Wall: Introduction to Virtual Special Issue on Pharmaceutical Cocrystals. Cryst. Growth Des..

[2] Good D., Rodriguez-Hornedo N. (2009). Solubility Advantage of Pharmaceutical Cocrystals. Cryst. Growth Des..

[3] Schultheiss N., Newman A. (2009). Pharmaceutical Cocrystals and Their Physicochemical Properties. Cryst. Growth Des..

[4] Miroshnyk I., Mirza S., Sandler N. (2009). Pharmaceutical co-crystals-an opportunity for drug product enhancement. Expert Opin. Drug Del..

[5] Lemmerer A., Bernstein J. (2011). Hydrogen Bonding Patterns of the Co-Crystal Containing the Pharmaceutically Active Ingredient Isoniazid and Terephthalic Acid. J. Chem. Crystallogr..

[6] Desiraju G. (1995). Supramolecular synthons in crystal engineering—a new organic synthesis. Angew. Chem. Int. Ed..

[7] Stuart M., Box K. (2005). Chasing Equilibrium: Measuring the Intrinsic Solubility of Weak Acids and Bases. Anal. Chem..

[8] Báthori N., Lemmerer A., Venter G., Bourne S., Caira M. (2011). Pharmaceutical Co-crystals with Isonicotinamide—Vitamin B3, Clofibric Acid, and Diclofenac—and Two Isonicotinamide Hydrates. Cryst. Growth Des..

[9] (2005). APEX2 v2.2.0.

[10] (1997). SAINT v7.46a.

[11] (2008). XPREP Version 2008/2 for Windows.

[12] (2008). SHELXTL v6.10.

[13] Allen F. (2002). The Cambridge Structural Database: a quarter of a million crystal structures and rising. Acta. Crystallogr. B.

[14] Aakeröy C., Salmon D. (2005). Building co-crystals with molecular sense and supramolecular sensibility. CrystEngComm.

[15] Etter M., Adsmond D. (1990). The use of cocrystallization as a method of studying hydrogen bond preferences of 2-aminopyrimidine. J. Chem. Soc. Chem. Commun..

[16] Etter M., Adsmond D., Britton D. (1990). 2-Aminopyrimidine-succinic acid (1/1) cocrystal. Acta Crystallogr. C.

[17] Etter M., Frankenbach G., Adsmond D. (1990). Using hydrogen bonds to design acentric organic materials for nonlinear optical users. Mol. Cryst. Liq. Cryst..

[18] Desiraju G., Parthasarathy R. (1989). The Nature of Halogen-Halogen Interactions: Are Short Halogen Contacts Due to Specific Attractive Forces or Due to Close Packing of Nonspherical Atoms?. J. Am. Chem. Soc..

[19] Aakeröy S., Forbes S., Desper J. (2009). Using Cocrystals to Systematically Modulate Aqueous Solubility and Melting Behavior of an Anticancer Drug. J. Am. Chem. Soc..

[20] Lynch D. (2009). Private Communication to the Cambridge Structural Database, version 5.32.

